# Massive vaginal bleeding caused by ruptured cervical varices associated with fetal loss in the second trimester of pregnancy: A report of a rare case

**DOI:** 10.3892/mi.2025.234

**Published:** 2025-04-10

**Authors:** Anna Thanasa, Efthymia Thanasa, Ioannis-Rafail Antoniou, Alexandros Leroutsos, Ioannis Thanasas

**Affiliations:** 1 Department of Health Sciences, Medical School, Aristotle University of Thessaloniki, 54124 Thessaloniki, Greece; 2 Department of Obstetrics and Gynecology, General Hospital of Trikala, 42100 Trikala, Greece

**Keywords:** cervical varices, placenta previa, vaginal bleeding, transvaginal Doppler ultrasound, fetal loss, caesarean section

## Abstract

Cervical varices during pregnancy are a rare clinical entity. The rupture of cervical varices in pregnant women may be associated with fetal loss following the termination of the pregnancy or pre-term delivery, in the case that it occurs in the presence of a viable fetus. The present study describes a case of pregnancy termination by caesarean section due to massive vaginal bleeding following the rupture of cervical varices in the second trimester. A 26-year-old primigravida, with a history of placenta previa, presented to the hospital at 21 weeks of gestation, reporting significant painless vaginal bleeding. A transvaginal Doppler ultrasonography revealed elongated formations in the endocervix with increased vascularization, suggestive of dilated vascular structures. There were no obvious signs of peripheral abruption of the placenta previa. In the operating room, a thorough examination of the cervix revealed enlarged blood vessels with a variceal appearance, protruding through the cervical canal to the external cervical os, which were actively bleeding. Upon palpation of the vascular structures, torrential vaginal bleeding ensued. After temporarily controlling the bleeding with the use of several hemostatic forceps, the termination of the pregnancy was performed via cesarean section. To manage the severe intraoperative bleeding, blood transfusion was administered, and an utero-cervico-vaginal tamponade was performed using gauze packing. Blood transfusions were also required in the immediate post-operative period to stabilize the hemodynamic condition of the patient. The patient was discharged from the clinic on the 5th post-operative day. At 20 days thereafter, a clinical examination and transvaginal Doppler ultrasonography revealed normal cervical findings. On the whole, the case described herein highlights the importance of transvaginal Doppler ultrasonography in the early diagnosis of cervical varices. It also underscores the need for a differential diagnosis between bleeding from cervical varices and vaginal bleeding caused by peripheral placental abruption, with the goal of minimizing maternal and perinatal morbidity and mortality.

## Introduction

Varices are dilated, edematous superficial veins located in the subcutaneous tissue, commonly occurring in pregnant women, and are typically found in the lower extremities. Pregnancy is considered a significant contributing factor to the increased incidence of varicose veins (1,2). In addition to the lower extremities, varicose veins can also appear in the hemorrhoidal plexus, vulva, vagina and, less commonly, in the cervix during pregnancy. The incidence of vulvar varicose veins in pregnant women exhibited an increase, affecting up to 8% of all pregnancies (3). The management of severe vulvovaginal varices presents a challenge in everyday obstetric practice, often requiring the collaboration of radiologists, vascular surgeons and obstetrician-gynecologists (4).

Cervical varices are a rare clinical finding during pregnancy. To date, only 21 cases have been documented in the international English-language literature (5). Cervical varices are dilated vascular structures that can protrude through the dilated endocervical canal to varying degrees. Their superficial position close to the epithelium and their dilation, renders them anatomical weak points, susceptible to rupture, thus potential causes of severe, life-threatening bleeding during the antenatal period (5). Their pathogenetic mechanisms are not yet fully understood; however, their incidence appears to be associated with low placental implantation, an advanced maternal age, twin pregnancies and exposure to diethylstilbestrol (5,6). Based on data from the literature, cervical varices may be superficial or originating from the endocervix, a feature that significantly affects appearance during a physical examination and imaging (7). Superficial, os-type varices are more easily visualized during a speculum examination, as is the detection of the precise site of bleeding in the case of rupture (5,7). Internal, endocervix varices are usually more difficult to detect via speculum examination, and are better visualized via vaginal ultrasound and Doppler mode (5,7). However they may also be visible via a speculum examination in the case of multiple, highly enlarged varices that protrude from the external os (such as in the case in the present study). Hemorrhagic endocervical varices are rarely observed in the first trimester of pregnancy. Cervical varices usually occur in the second or third trimester of pregnancy and may be associated with fetal loss following the termination of the pregnancy with a non-viable fetus (as in the case in the present study) or with pre-term delivery (6).

The present study describes a rare case of fetal loss at 21 weeks of gestation due to severe bleeding from ruptured cervical varices. The authors wish to emphasize the role of transvaginal ultrasonography and transvaginal Doppler ultrasonography in the early diagnosis of this condition, aiming to reduce maternal and perinatal morbidity and mortality. Additionally, the importance of differentiating bleeding caused by cervical varices from vaginal bleeding associated with peripheral placental abruption, particularly in cases complicated by placenta previa is highlighted.

## Case report

The present case report concerns a 26-year-old primigravida who presented early in the morning to the Emergency Department of the General Hospital of Trikala, Trikala, Greece, at 21 weeks of gestation, reporting an episode of major vaginal bleeding at ~1 h prior. The pregnant woman described the bleeding as heavy, with large blood clots. Her hemodynamic status was stable, with a blood pressure of 110/70 mmHg and a heart rate of 84 beats per minute, indicating tolerable blood loss severity and allowing for the performance of all necessary investigations. The vaginal hemorrhage was not accompanied by pain. The patient had been followed-up at a private obstetric center, where a low-lying placenta (placenta previa) was identified via ultrasound during the first trimester. Nuchal translucency was measured at 1.2 mm, and no fetal anatomical abnormalities were detected. A previous minor vaginal hemorrhage (first episode) was reported ~1 month earlier. According to the patient, the bleeding, attributed by her obstetrician to peripheral abruption of the placenta previa, resolved within 1 week following bed rest and oral progesterone administration. An investigation of her medical history did not reveal any notable findings, and no evidence of exposure to diethylstilbestrol was found. This was a singleton pregnancy, resulting from spontaneous conception.

**Figure 1:**
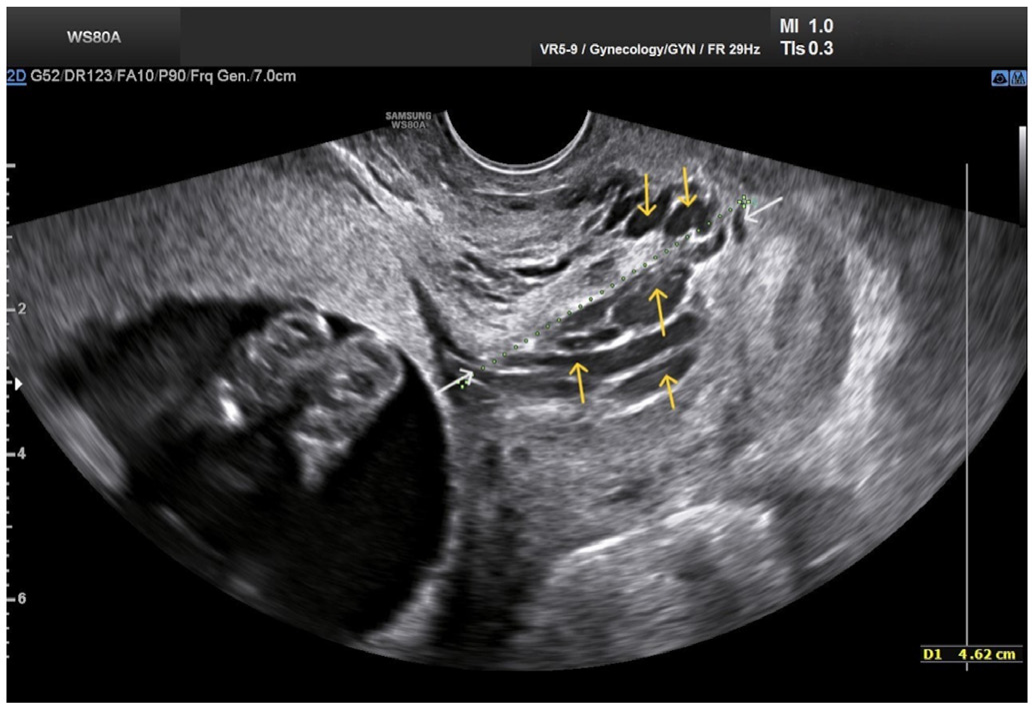
Transvaginal ultrasound imaging of cervical varices in the second trimester of pregnancy: The visualization of dilated longitudinal structures (yellow arrows) protruding through the enlarged endocervix is evident. The white arrows indicate the internal cervical os (left) and the external cervical os (right).

**Figure 2:**
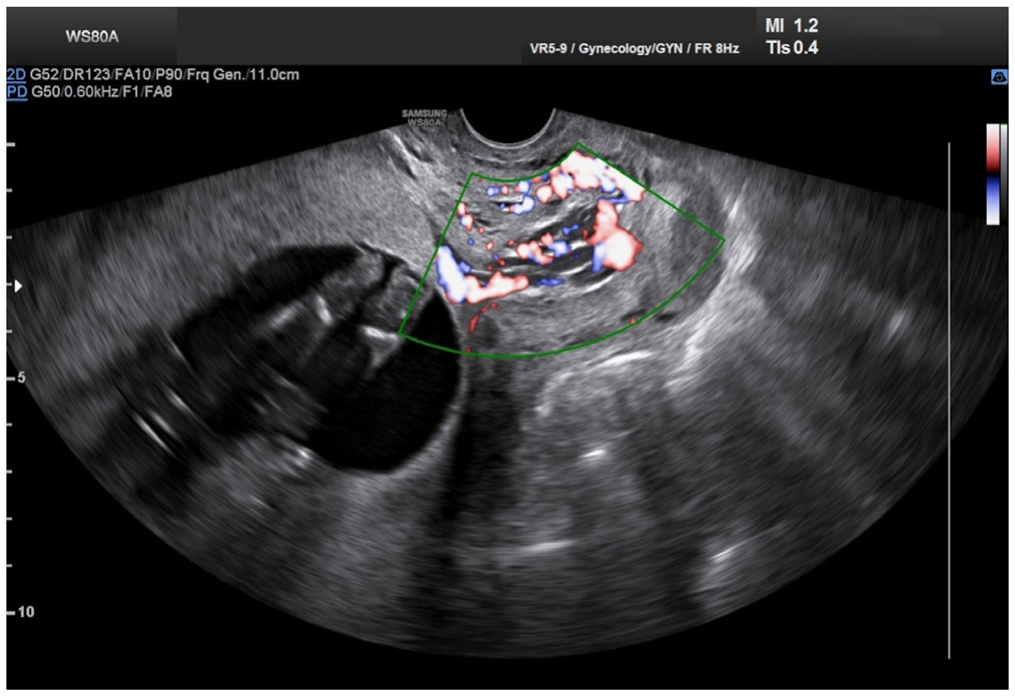
Transvaginal Doppler ultrasound imaging of cervical varices in the second trimester of pregnancy: Longitudinal dilated structures with increased vascularity are visualized within the endocervix, involving the majority of the cervix, suggesting dilated vascular structures.

**Figure 3:**
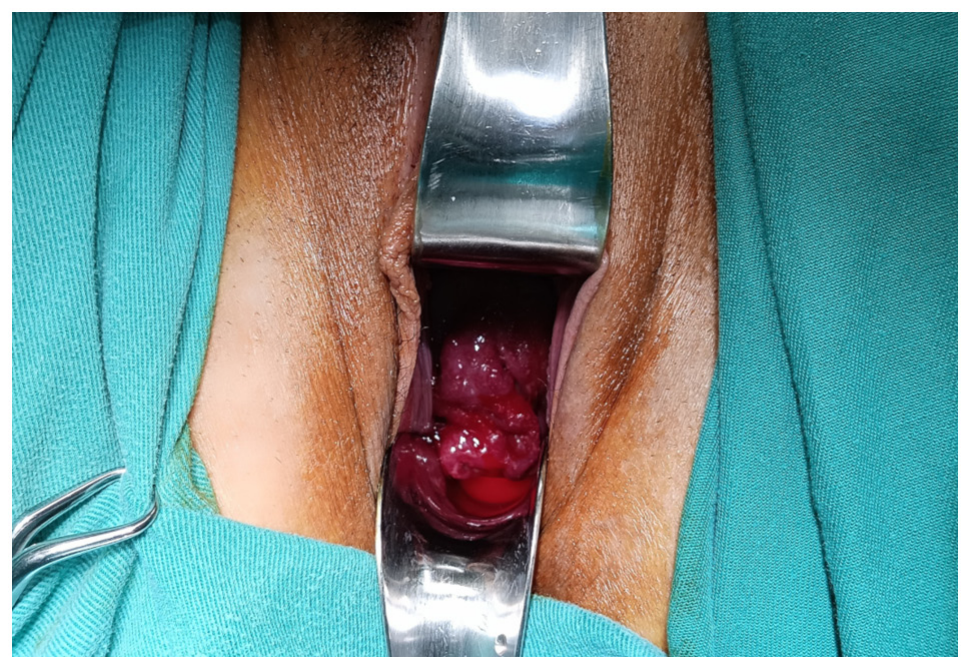
Clinical examination of cervical varices in the second trimester of pregnancy: Upon examination, a mass of large, dilated vessels with a varicose configuration protruding from the external cervical os and actively bleeding was identified.

**Figure 4:**
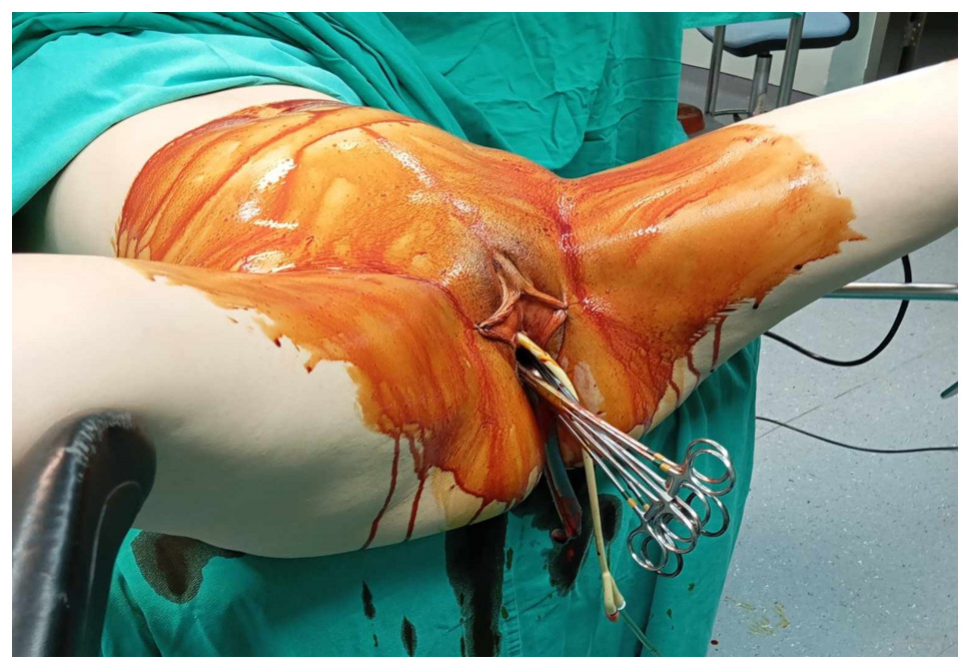
Rupture of cervical varices in the second trimester of pregnancy: In the operating room, under general anesthesia, while attempting to palpate the cervical varices, torrential vaginal hemorrhage was triggered by their rupture. For temporary management, several hemostatic forceps were applied to arrest the bleeding.

Upon a gynecological vaginal examination, a large amount of vaginal bleeding was observed, which obscured a clear view of the cervix during the examination. An obstetric ultrasound revealed a normal fetal heart rate, with the amount of amniotic fluid within normal limits. The placenta was located in an anterior low position, with no obvious signs of abruption on the ultrasound. The transvaginal ultrasound revealed a normal cervical length of 46 mm. However, the imaging revealed dilated, elongated structures protruding through the dilated endocervical canal, which were characteristic (Fig. 1). A transvaginal Doppler ultrasound of the endocervix demonstrated dilated, elongated longitudinal structures with increased vascularization, involving the majority of the cervix. These findings were consistent with dilated vascular structures (Fig. 2). Due to the ongoing vaginal bleeding following the admission of the patient to the clinic, a more thorough vaginal examination was performed under general anesthesia in the operating room. The operating theatre was adequately prepared for the possibility of pregnancy termination via laparotomy in the event of a major hemorrhage. A careful inspection of the cervix revealed enlarged varicose-like vessels projecting through the cervical canal up to the external cervical os, which were actively bleeding (Fig. 3). During an attempt to palpate the vascular structures in the cervix, profuse vaginal bleeding was triggered. To manage the bleeding temporarily, several hemostatic forceps were applied (Fig. 4).

**Figure 5:**
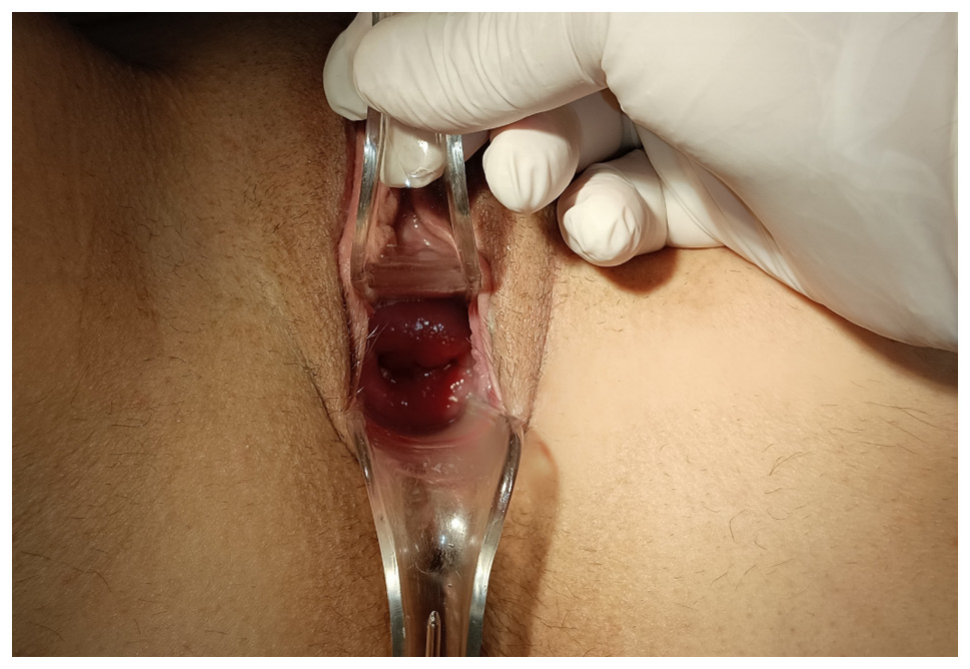
Clinical examination of the cervix 20 days following fetal loss: Normal findings were observed. The cervical margins are clearly visible.

**Figure 6:**
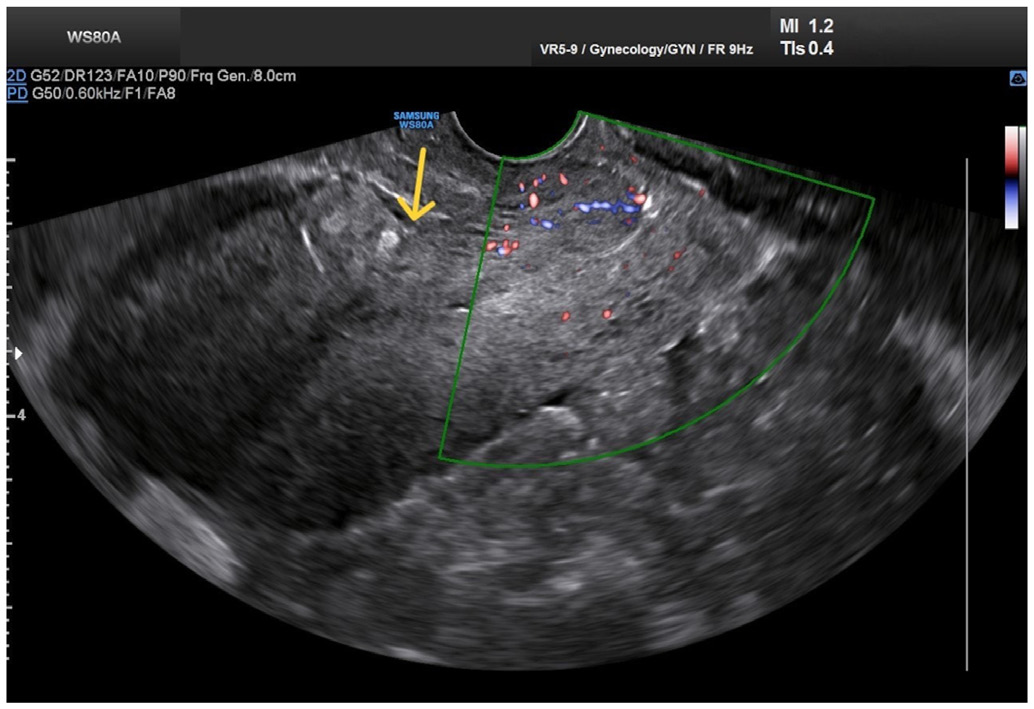
Transvaginal Doppler ultrasound imaging of the cervix 20 days after fetal loss: The findings are normal, with the cesarean scar healing site in the uterus (yellow arrow) appearing intact and without abnormalities.

With the patient in the gynecological laparoscopy position, an emergency cesarean section was performed. The umbilical vessels were clipped and the fetus was delivered and subsequently handed to the neonatologist for assessment, who unfortunately confirmed its demise shortly thereafter. The placenta was removed, without any evidence of placenta accreta spectrum disorder or vascular malformations in the lower uterine segment near the internal cervical os being observed. However, severe intraoperative bleeding necessitated the decision to perform a utero-cervico-vaginal tamponade with gauze packing, along with the transfusion of 3 units of packed red blood cells and 1 unit of fresh frozen plasma. Prior to the tamponade, the previously placed hemostatic forceps were removed. Immediately post-operatively, the dilated vessels in the endocervix were no longer visible, and no active bleeding was observed during inspection. For post-operative hemodynamic stabilization (Table I), an additional transfusion of 3 units of packed red blood cells was required. The utero-cervico-vaginal gauze tamponade was removed on the first post-operative day. Antibiotic treatment was initiated, with cefuroxime (Mefoxil) administered at a dose of 2 g every 8 h for 4 days, combined with metronidazole (Flagyl) at 500 mg every 8 h for 2 days. The patient was discharged from the clinic on the 5th post-operative day. At 20 days thereafter, both the clinical cervical examination findings (Fig. 5) and transvaginal Doppler ultrasound findings (Fig. 6) were normal. Since then, all subsequent follow-up assessments have been performed at the private obstetric center the patient attended before her presentation to our hospital. At the time of writing the patient is healthy, without bleeding and has not yet made another attempt at conception and pregnancy.

## Discussion

The pathogenesis of cervical varices in pregnant women has not been fully elucidated. Placenta previa is thought to be the main risk factor. Placenta previa, along with the increased cervical blood flow that characterizes such cases, appears to be significantly associated with the development of venous distension and varices in the area (7). In addition, prenatal maternal exposure to diethylstilbestrol, which can cause vascular malformations in the pelvic organs, has been implicated in the development of cervical varicose veins. Moreover, the interaction between the uterus and placenta in women exposed to diethylstilbestrol is well known (8). Furthermore, *in vitro* fertilization, the multiple pregnancies frequently resulting from it, and a maternal age >35 years, which often characterizes these pregnant women, are additional risk factors for cervical varices (9). In rare cases, the development of cervical varices in pregnancy may occur in the absence of these risk factors (10). The patient in the present study was 26 years of age and carried a singleton pregnancy following spontaneous conception. No evidence suggesting exposure to diethylstilbestrol at the time of the case was found. In the patient described herein, the only predisposing risk factor for the development of cervical varices was a low-lying placenta.

The prenatal diagnosis of cervical varices is based on a clinical examination, transvaginal ultrasound and magnetic resonance imaging. The recognition of the clinical features of cervical varices during pregnancy is of utmost importance for the accurate diagnosis of this rare clinical entity before major bleeding occurs due to rupture (11). Bleeding is the predominant symptom and usually indicates rupture of the cervical varices (12). It is crucial to avoid confusion with vaginal hemorrhage due to peripheral abruption of a placenta previa, which often accompanies cervical varices (5). Additionally, the differentiation between bleeding caused by the rupture of cervical varices and that caused by vasa previa is of great diagnostic value. Vasa previa originate from the placenta, whereas cervical varices are of maternal origin and are not necessarily related to the placenta (12). A gynecological clinical examination with careful cervical inspection is thought to be of considerable help in the diagnosis of cervical varices. However, unlike transvaginal ultrasound and magnetic resonance imaging, it is unable to accurately assess the origin, nature, and extent of cervical varices (12).

Transvaginal ultrasound easily detects the presence of a placenta previa, which often accompanies cervical varices in pregnant women. At the same time, it can identify a hypoechoic structure in the endocervix, which, when examined by Doppler transvaginal ultrasound, suggests a vascular origin (13). Magnetic resonance imaging, although not readily available for every pregnant woman with bleeding, is considered very valuable for accurately visualizing the vessel distribution in relation to adjacent organs and for excluding other unforeseen pelvic pathological conditions prior to a planned cesarean section (12). In the patient in the present study, the presence of placenta previa and the onset of a small vaginal hemorrhage in the late first trimester initially raised the suspicion of possible peripheral placental abruption. The diagnosis of cervical varices was established by transvaginal Doppler ultrasonography and a detailed vaginal examination under anesthesia after the incident of severe vaginal bleeding in the second trimester. Upon a cervical examination, a mass of dilated longitudinal vessels with a variceal configuration was found, originating from the endocervix and protruding up to the external cervical os, extending mainly toward the posterior cervical margin (Fig. 3).

Scientific evidence supporting the optimal management of bleeding caused by cervical varices in pregnancy is still lacking. The limitation of physical activity, bed rest, avoidance of sexual intercourse and blood transfusions in cases where bleeding is accompanied by symptoms of anemia, such as weakness, orthostatic hypotension, dizziness, or dyspnea are common conservative therapeutic interventions (12). Additionally, the application of a cervical pessary is another conservative option to control bleeding associated with cervical varices during pregnancy. It is believed that the pressure exerted by the pessary on the cervical tissues may reduce the width of the varices, thus decreasing the bleeding caused by their rupture (14). Furthermore, cervical cerclage can be successfully applied to treat cervical varicose veins in pregnant women and can lead to a successful full-term pregnancy, even when applied in cases of active bleeding from the rupture of cervical varices, as performed by Poliektov and Kahn (5). The case described herein differs in the fact that there was no time to perform conservative measures, since massive bleeding was triggered only by the attempt to examine the cervix. It is very likely that any operative maneuvers in the area, such as cervical cerclage, would also trigger massive bleeding. In cases where a decision is made to terminate the pregnancy in the first or second trimester, prophylactic embolization of the uterine artery is considered to markedly reduce the risk of bleeding from cervical varices, such as the case described by Lesko *et al* (15). In the patient in the present study, the decision to terminate the pregnancy was an emergency medical option due to massive vaginal bleeding caused by palpation of the cervical varices. The severe vaginal bleeding in the case in the present study left no room for consideration of conservative methods for managing cervical varices, which may have allowed for the continuation of the pregnancy and its termination by planned cesarean section or vaginal delivery. The management course used herein is similar to the one followed in the study by Kumazawa *et al* (16), who managed their case by mechanical pressure of the bleeding varices via vaginal packing. In their case, mechanical pressure was effective in stopping the bleeding and prolonging pregnancy for a few more days, with emergency caesarean section being ultimately performed (16), similar to the case described herein. However, the case in the present study differs, as it demonstrates a more clinically challenging scenario, whereby the bleeding from varicose veins could not be controlled via mechanical pressure, leading to the performance of emergency caesarean section earlier during gestation and allowing no margins to attempt other conservative treatment options to control the bleeding.

A planned cesarean section is still the preferred mode of delivery for pregnant women with cervical varices. A planned cesarean section avoids the rupture of cervical varices and the triggering of massive vaginal bleeding that can occur during vaginal delivery (17). A planned vaginal delivery may be appropriate in isolated cases of cervical varices, in which the varices may resolve simultaneously with the separation of a coexisting placenta previa (6,18). Emergency cesarean section, either associated with or without emergency hysterectomy, is indicated in cases of massive, uncontrolled bleeding and is associated with significantly increased rates of maternal and perinatal morbidity and mortality (19).

The prognosis is favorable in asymptomatic pregnant women without bleeding from ruptured cervical varices. Following the delivery of the fetus, cervical varices resolve spontaneously, and the risk of recurrence is considered to be low (12). In cases where the rupture of cervical varices occurs, the bleeding is severe and life-threatening for both the pregnant woman and the newborn (20). In the patient described herein, the profuse vaginal bleeding caused by rupture of the cervical varices resulted in the loss of the fetus after the pregnancy was terminated by performing a cesarean section. In both the immediate and late post-operative periods, the findings from clinical examination and transvaginal Doppler ultrasonography were normal.

The primary strength of the present case report is its rarity in the clinical setting, thus its addition to the literature provides further insight into a lesser known clinical entity and its management. However, there are limitations that need to be acknowledged. Namely, the availability of only a single case somewhat limits the generalizability and applicability of our findings and conclusions in different clinical settings. Finally, the absence of long-term follow-up, particularly up to a second pregnancy and the exploration of recurrence is an additional limitation that should be acknowledged. The individualization of diagnostic and treatment approach based on the circumstances of each patient and the available resources and expertise at hand is key in achieving the most favorable outcomes.

In conclusion, cervical varices are an extremely rare clinical entity. The rupture of cervical varices, leading to massive vaginal bleeding in the second trimester of pregnancy, often necessitates the termination of the pregnancy via cesarean section. Cervical varices, particularly when associated with placenta previa, should be considered in the differential diagnosis of painless vaginal bleeding in pregnant women. The careful and skilled use of transvaginal Doppler ultrasonography is deemed to be invaluable for the early and accurate diagnosis of cervical varices. Early detection can prevent unnecessary diagnostic procedures, such as the digital palpation of the cervix, which may otherwise result in variceal rupture, severe hemorrhage and fetal loss.

## Figures and Tables

**Table I:** Pre-operative and post-operative laboratory tests of the patient during her hospitalization at the clinic.

Laboratory tests	Pre-operatively	At 6 h after surgery	1st post-operative day	2nd post-operative day	4th post-operative day	Normal laboratory values
Ht	26.6%^a,b^	20.8%^c^	18.2%^d^	25.4%	25.4%	37.7-49.7%
Hb	9.7 g/dl	7.6 g/dl	6.7 g/dl	9.2 g/dl	8.6 g/dl	11.8-17.8 g/dl
WBC	9.1x10^3^/ml	16.1x10^3^/ml	14.4x10^3^/ml	13.89x10^3^/ml	7.51x10^3^/ml	4-10.8x10^3^/ml
NEUT	83.2%	87.3%	86.8%	78.7%	62.1%	40-75%
PLT	205x10^3^/ml	121x10^3^/ml	107x10^3^/ml	135x10^3^/ml	186x10^3^/ml	150-350x10^3^/ml
CRP	0.06 mg/dl	0.3 mg/dl	5.55 mg/dl	14.55 mg/dl	2.17 mg/dl	<0.5 mg/dl
APTT	29.8 sec	27.8 sec	35.3 sec	31 sec	31 sec	24.0-35.0 sec
INR	0.89	0.97	1.04	1.02	1.02	0.8-1.2
FIB	286 mg/dl	232 mg/dl	237 mg/dl	422 mg/dl	385 mg/dl	200-400 mg/dl
Glu	76 mg/dl	133 mg/dl	69 mg/dl	81 mg/dl	90 mg/dl	75-115 mg/dl
U	30 mg/dl	29 mg/dl	22 mg/dl	25 mg/dl	24 mg/dl	10-50 mg/dl
Cr	0.6 mg/dl	0.6 mg/dl	0.46 mg/dl	0.51 mg/dl	0.49 mg/dl	0.40-1.10 mg/dl
K^+^	4.17 mmol/l	4.1 mmol/l	3.24 mmol/l	3.51 mmol/l	3.49 mmol/l	3.5-5.1 mmol/l
Na^+^	135 mmol/l	133 mmol/l	140.5 mmol/l	139 mmol/l	141.7 mmol/l	136-145 mmol/l
B	0.5 mg/dl	0.92 mg/dl	0.48 mg/dl			0.3-1.2 mg/dl
SGOT	28 IU/l	27 IU/l	17 IU/l			5-33 IU/l
SGPT	13 IU/l	14 IU/l	11 IU/l			10-37 IU/l

^a^Ht and Hb values correspond to 3 h after the reported incident of acute vaginal bleeding. In previous laboratory tests conducted 1 week ago, the values were: 33.2%, Hb=11.6 g/dl;

^b^the transfusion of 1 unit of packed red blood cells was started pre-operatively, followed by the transfusion of 2 units of packed red blood cells;

^c^transfusion of 1 unit of packed red blood cells;

^d^transfusion of 1 unit of packed red blood cells. Ht, hematocrit; Hb, hemoglobin; WBC, white blood cells; NEUT, neutral; PLT, platelets; CRP, C-reactive protein; APTT, activated partial thromboplastin time; INR, international normalized ratio; FIB, fibrinogen; Glu, glucose; U, urea; Cr, creatinine; K^+^, potassium; Na^+^, sodium; B, bilirubin; SGOT, serum glutamic oxaloacetic transaminase; SGPT, serum glutamate pyruvate transaminase.

## Data Availability

The data used in the current study are available from the corresponding author upon reasonable request.

## References

[1] Raetz J, Wilson M, Collins K (2019). Varicose veins: Diagnosis and treatment. Am Fam Physician.

[2] Ismail L, Normahani P, Standfield NJ, Jaffer U (2016). A systematic review and meta-analysis of the risk for development of varicose veins in women with a history of pregnancy. J Vasc Surg Venous Lymphat Disord.

[3] Gavrilov SG (2017). Vulvar varicosities: Diagnosis, treatment, and prevention. Int J Womens Health.

[4] Giannella L, Montanari M, Delli Carpini G, Di Giuseppe J, Ciavattini A (2022). Huge vulvar varicosities in pregnancy: Case report and systematic review. J Int Med Res.

[5] Poliektov N, Kahn BF (2022). Bleeding cervical varices in pregnancy: A case report and review of the literature. J Neonatal Perinatal Med.

[6] Wax JR, Cartin A, Litton C, Conroy K, Pinette MG (2018). Cervical varices: An unusual source of first-trimester hemorrhage. J Clin Ultrasound.

[7] Tanaka M, Matsuzaki S, Kumasawa K, Suzuki Y, Endo M, Kimura T (2016). Cervical varix complicated by placenta previa: A case report and literature review. J Obstet Gynaecol Res.

[8] Thorp JM Jr, Fowler WC, Donehoo R, Sawicki C, Bowes WA Jr (1990). Antepartum and intrapartum events in women exposed in utero to diethylstilbestrol. Obstet Gynecol.

[9] Yoshimura K, Hirsch E, Kitano R, Kashimura M (2004). Cervical varix accompanied by placenta previa in twin pregnancy. J Obstet Gynaecol Res.

[10] Youssef J, Afolayan V, Mack M, Sze A (2023). Cervical varicosities, an uncommon cause of third-trimester bleeding in pregnancy: A case report. Case Rep Womens Health.

[11] Kurihara Y, Tachibana D, Teramae M, Matsumoto M, Terada H, Sumi T, Koyama M, Ishiko O (2013). Pregnancy complicated by cervical varix and low-lying placenta: A case report. Jpn Clin Med.

[12] Peng MY, Ker CR, Lee YS, Ho MC, Chan TF (2018). Cervical varices unrelated to placenta previa as an unusual cause of antepartum hemorrhage: A case report and literature review. Taiwan J Obstet Gynecol.

[13] Kusanovic JP, Soto E, Espinoza J, Stites S, Gonçalves LF, Santolaya J, Nien JK, Erez O, Sorokin Y, Romero R (2006). Cervical varix as a cause of vaginal bleeding during pregnancy: Prenatal diagnosis by color Doppler ultrasonography. J Ultrasound Med.

[14] González-Bosquet E, Grau L, Ferrero-Martínez S, Hernandez-Saborit A, Rebollo M, Gomez-Chiari M, Martínez Crespo JM, Gómez-Roig MD (2021). Pessary for management of cervical varices complicating pregnancy. Obstet Gynecol.

[15] Lesko J, Carusi D, Shipp TD, Dutton C (2014). Uterine artery embolization of cervical varices before second-trimester abortion. Obstet Gynecol.

[16] Kumazawa Y, Shimizu D, Hosoya N, Hirano H, Ishiyama K, Tanaka T (2007). Cervical varix with placenta previa totalis. J Obstet Gynaecol Res.

[17] Sammour RN, Gonen R, Ohel G, Leibovitz Z (2011). Cervical varices complicated by thrombosis in pregnancy. Ultrasound Obstet Gynecol.

[18] Wong CK, Hung CMW, Ng VKS, Yung WK, Leung WC, Lau WL (2022). Four cases of cervical varices without placenta praevia: Presentation, diagnosis, managements, and literature review. J Obstet Gynaecol Res.

[19] Saedi N, Ghaemi M, Moghadam M, Haddadi M, Hashemi Z, Hantoushzadeh S (2023). Emergency postpartum hysterectomy as a consequence of cervical varix during pregnancy; a case report and literature review. Int J Surg Case Rep.

[20] Park JE, Kim MJ, Kim MK, Kim HM (2019). Cervical varix with thrombosis diagnosed in the first trimester of pregnancy. Obstet Gynecol Sci.

